# Medical Sciences section of The Journals of Gerontology Series A Editor’s Choice Articles of 2024: The Effect of Life Experiences on Aging and Disease.

**DOI:** 10.1093/geroni/igaf122.1350

**Published:** 2025-12-31

**Authors:** Lewis Lipsitz, Raya Kheirbek

## Abstract

This symposium will present four 2024 “Editor’s Choice” articles from the Medical Sciences section of The Journals of Gerontology, Series A: Biological Sciences and Medical Sciences that focus on the effect of life experience and life-style influences on aging and disease. Maddison Mellow and her colleagues, in their article “Intensity and Domain-Based 24-Hr Activity Patterns Differ in Their Links to Cognitive and Cardiometabolic Health” examined how differences in the intensity and timing of physical activities can influence cognitive function and cardiometabolic health in older adults. So Yeon Jeon and her coauthors, in “Caregiving-Related Depression Increases Neuroinflammation in older adult spousal caregivers to Individuals With Cognitive Impairment: A Longitudinal Study” look at the neurobiological impact of caregiving to individuals with cognitive impairments and how it contributes to neuroinflammation, a well-known risk factor for various diseases. In “Sociodemographic Determinants of Extreme Heat and Ozone Risk Among Older Adults in 3 Sun Belt Cities”, Peter Crank and colleagues surveyed how older adults are affected by extreme heat and ozone pollution inside and outside of their homes across select cities in the United States. Christopher Kaufmann and his co-authors investigated the association between previous incarceration and various chronic health conditions in older adults in “Links of Previous Incarceration With Geriatric Syndromes and Chronic Health Conditions Among Older Adults in the United States”. Raya Kheirbek, our discussant, will draw upon her own research studying the unique needs of marginalized and medically vulnerable individuals to highlight commonalities and lessons learned from these studies.

